# Clinical outcomes and adverse events associated with intravenous polymyxin-containing therapy in hospitalized children with resistant Gram-negative infections: a systematic review and meta-analysis

**DOI:** 10.3389/fped.2026.1928217

**Published:** 2026-09-21

**Authors:** Huilian Chen, Mure Ali, Hongyuan Huang, Junjie Ning, Shuanghong Luo, Lina Qiao

**Affiliations:** 1 Pediatric Department, West China Second University Hospital, Sichuan University, Chengdu, China; 2 NHC Key Laboratory of Chronobiology, Sichuan University, Chengdu, China; 3 School of Medical and Life Sciences, Chengdu University of Traditional Chinese Medicine, Chengdu, China

**Keywords:** colistin, hospitalized children, nephrotoxicity, pediatric intensive care, polymyxin B, polymyxins, resistant Gram-negative infections

## Abstract

**Background:**

Intravenous polymyxins remain salvage agents for resistant Gram-negative infections in children, but pediatric evidence on clinical outcomes and toxicity is fragmented.

**Methods:**

We systematically searched PubMed, Embase, Web of Science, the Cochrane Library, CNKI, Wanfang Data, SinoMed, and VIP from inception to 31 January 2025. Eligible studies reported intravenous polymyxin-containing therapy in hospitalized pediatric patients with documented or suspected resistant Gram-negative infections. Random-effects single-arm meta-analyses were used to estimate pooled outcome proportions.

**Results:**

Twenty-four studies comprising 1,152 reported patients or treatment units were included, most of which were single-arm observational studies. The pooled proportions were 22% [95% confidence interval (CI) 16%–27%] for all-cause mortality, 14% (95% CI 11%–17%) for infection-related mortality, 65% (95% CI 58%–72%) for clinical improvement, 13% (95% CI 9%–17%) for overall adverse events, and 10% (95% CI 7%–14%) for nephrotoxicity. Neurotoxicity and hepatotoxicity were infrequently reported. Clinical setting, dosing, outcome definitions, and units of analysis were heterogeneous.

**Conclusions:**

The available evidence describes outcomes observed during intravenous polymyxin-containing therapy in hospitalized children but does not establish comparative efficacy or causality for adverse events. Prospective multicenter pediatric studies using standardized dosing, outcome, and safety definitions are needed.

## Introduction

Polymyxins were first isolated in the 1940s from *Bacillus polymyxa*. Early studies demonstrated their potent antibacterial activity against various Gram-negative bacteria, particularly when other antibiotics proved ineffective. Consequently, they have been in clinical use since the 1950s (1). However, due to the potential risks of nephrotoxicity and neurotoxicity, polymyxins were gradually replaced by safer antibiotics (such as aminoglycosides and third-generation cephalosporins) in the 1970s. In recent years, with the rise of drug-resistant strains—particularly multidrug-resistant Gram-negative bacteria (MDR-GNB) such as *Pseudomonas aeruginosa* and *Acinetobacter baumannii*—polymyxins have regained clinical attention and been reintroduced into therapeutic use.

This resurgence is especially relevant in pediatric populations, where children receive antibiotics more frequently than any other class of drugs due to conditions ranging from common urinary tract infections to rare diseases such as meningitis. Consequently, the incidence and complexity of multidrug-resistant infections in children are increasing. As a last-line treatment for MDR-GNB infections, the use of polymyxins has also risen in this population (2, 3). However, as an older class of antibiotics with significant nephrotoxic and neurotoxic potential, polymyxins must be used cautiously in hospitalized pediatric patients, particularly in high-acuity inpatient settings (4).

Polymyxins are lipopeptide antibiotics that exert bactericidal activity against Gram-negative bacilli by disrupting bacterial cell membranes through electrostatic and hydrophobic interactions. The positively charged polymyxin molecules interact with negatively charged phosphate groups on the lipid A moiety of lipopolysaccharide (LPS) in the outer membrane of Gram-negative bacilli, thereby destabilizing the outer membrane. This instability enables polymyxin molecules to insert their hydrophobic regions into the outer membrane, causing further membrane disruption and facilitating additional polymyxin uptake (5, 6). Polymyxins exhibit a unique mechanism of action involving disruption of the outer membrane integrity in Gram-negative bacteria. In addition to providing rapid bactericidal activity, they can potentiate the activity of other antibiotic classes. By displacing divalent cations such as magnesium and calcium from the bacterial outer membrane, polymyxins increase membrane permeability, leading to cell content leakage and bacterial death (7). Based on their chemical structures, polymyxins are classified into polymyxin B and polymyxin E (also known as colistin). Although both share similar antibacterial spectra and clinical applications, Polymyxin E is more commonly used in clinical practice.

Pediatric evidence remains limited by small samples, predominantly retrospective designs, heterogeneous dosing, and inconsistent definitions of clinical response and nephrotoxicity. The available literature also extends beyond the pediatric intensive care unit to include burn, hematology/oncology, and mixed inpatient settings. By including evidence identified through English- and Chinese-language databases, evaluating both colistin/colistimethate sodium (CMS) and polymyxin B, and explicitly distinguishing patient-level from treatment-level reporting, this systematic review and meta-analysis aimed to summarize the observed clinical outcomes and adverse events associated with intravenous polymyxin-containing therapy in hospitalized children with resistant Gram-negative infections.

## Materials and methods

This systematic review and meta-analysis was conducted and reported in accordance with the PRISMA 2020 statement (8). The completed PRISMA 2020 checklist is provided in Supplementary File 1. The protocol was registered in the International Prospective Register of Systematic Reviews (PROSPERO; CRD42020173233).

### Search strategy

We systematically searched PubMed, Embase, Web of Science, the Cochrane Library, CNKI, Wanfang Data, SinoMed, and VIP from inception to 31 January 2025. The search strategy combined terms related to children and polymyxins/colistin. Reference lists of eligible studies and relevant reviews were also screened manually. Complete database-specific search strategies are provided in Supplementary File 2.

### Study selection

The eligibility criteria were structured according to the population, intervention, comparator, outcomes, and study design (PICOS) framework. The population was hospitalized pediatric patients with documented or highly suspected resistant Gram-negative infections. The intervention was intravenous polymyxin-containing therapy, including colistin/colistimethate sodium or polymyxin B. A comparator was not required because most eligible studies were single-arm observational studies; when comparative data were available, comparator group information was extracted descriptively. Outcomes included all-cause mortality, infection-related mortality, clinical improvement, microbiological eradication, overall adverse events, nephrotoxicity, neurotoxicity, and hepatotoxicity. Eligible study designs included randomized trials, comparative cohort studies, and observational case series with extractable pediatric outcome data.

Studies were eligible if they enrolled hospitalized pediatric patients or provided extractable pediatric subgroup data for intravenous polymyxin treatment. Eligible settings included pediatric intensive care units and other high-acuity inpatient settings, such as burn units, hematology/oncology wards, and mixed hospital wards. Studies of documented multidrug-resistant (MDR), extensively drug-resistant (XDR), carbapenem-resistant Gram-negative infections, or highly suspected resistant Gram-negative infections treated with intravenous polymyxins were eligible. To reflect the reporting practice of the original literature, both patient-level and treatment-level analyses (including episodes, courses, and indications) were considered eligible.

The following studies were excluded: 1) studies conducted exclusively in cystic fibrosis patients, because polymyxin use in cystic fibrosis often reflects chronic airway colonization, recurrent pulmonary exacerbations, or inhaled/long-term antimicrobial strategies, which differ clinically from acute resistant Gram-negative infections requiring intravenous salvage therapy in hospitalized children; 2) studies not reporting treatment outcomes or adverse events of intravenous polymyxins; 3) studies involving polymyxins used solely for prophylaxis, decontamination, or diarrhea treatment; 4) studies involving only topical, oral, intraventricular, or intrathecal administration without extractable intravenous treatment data; 5) reviews, letters, abstracts without sufficient original data for extraction, and duplicate publications; and 6) studies with insufficient data for outcome synthesis. Intravenous polymyxin therapy had to be a main treatment component; adjunctive non-intravenous administration was allowed provided that intravenous treatment outcomes were reported for the included cohort.

Two reviewers independently screened titles and abstracts, and then assessed potentially eligible full-text reports. Disagreements were resolved by discussion and, when necessary, consultation with a third reviewer.

### Risk-of-bias assessment

Risk of bias was assessed according to study design. The randomized controlled trial was evaluated using the Cochrane RoB 2 tool (9). Comparative retrospective cohort studies were assessed using the JBI Critical Appraisal Checklist for Cohort Studies (10). Single-arm retrospective or prospective observational studies without an external comparator were assessed using the JBI Critical Appraisal Checklist for Case Series (11). Two investigators independently performed the quality assessment, and any disagreements were resolved through discussion with a third investigator.

### Outcomes and definitions

The primary outcome measure was all-cause mortality among hospitalized pediatric patients receiving intravenous polymyxin therapy for resistant Gram-negative bacterial infections. Infection-related mortality was also recorded when available. Secondary outcomes included 1) the number and type of adverse events, 2) clinical cure/improvement, and 3) microbiological eradication defined as clearance of the original pathogen in follow-up cultures. Nephrotoxicity and neurotoxicity were evaluated based on the definitions used in each included study. Because the included studies used different nephrotoxicity definitions, we extracted the definition used in each study when available. Reported definitions included serum creatinine elevation, acute kidney injury criteria, risk, injury, failure, loss of kidney function, and end-stage kidney disease (RIFLE)-based definitions, or author-defined renal adverse events. When no explicit definition was provided, nephrotoxicity was recorded according to the renal adverse-event label reported by the original study.

### Data extraction

Two investigators independently completed the data extraction and cross-checked the results. Any discrepancies were resolved by a third investigator. Extracted data included first author and year, study type, country, age, setting, analyzed unit (patients, episodes, courses, or indications), pathogens, route of administration, drug and dosage, average duration of therapy, outcomes, and adverse effects.

### Statistical analysis

We analyzed the proportional data using a random-effects model with arcsine transformation to calculate the overall mortality rate and its corresponding 95% confidence interval (CI) (12, 13). We assessed statistical heterogeneity using the *I*^2^ statistic (14). Prespecified subgroup analyses were performed according to polymyxin dose, pathogen category, and treatment duration. Publication bias was assessed by funnel plot inspection and Egger's test, and trim-and-fill analysis was applied when asymmetry was detected. Because the original literature variably reported patients, treatment episodes, courses, and indications, pooled estimates were interpreted according to the reporting unit of the source studies. Statistical analyses were conducted using Stata 15.0. Subgroup estimates were interpreted descriptively because formal tests for interaction were not reported in the available analysis. Study-specific confidence intervals in the forest plots are displayed on the raw proportion scale; intervals extending below 0 or above 1 are truncated at the plotting boundary for visualization only.

## Results

### Literature search

In the study selection process (Figure 1), we screened 3,302 unique records. After screening titles and abstracts, 215 full-text articles were assessed for eligibility. Of these, 24 studies met the inclusion criteria (15–38). The study selection process is presented in Figure 1. Most included studies were retrospective or prospective single-arm observational studies, while two studies were comparative retrospective cohorts and one was a randomized controlled trial.

**Figure 1:**
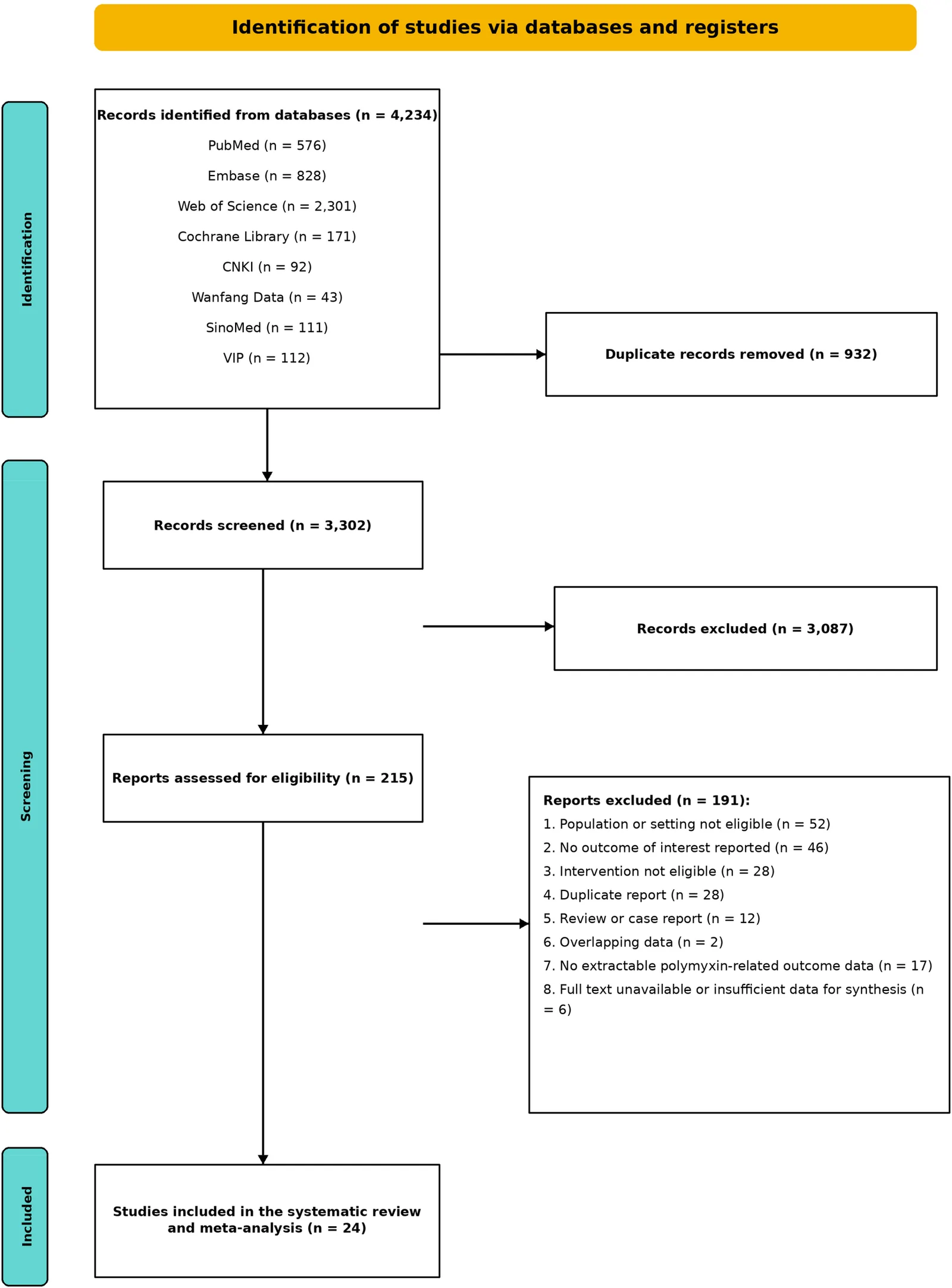
PRISMA flow diagram of study selection.

### Risk-of-bias assessment

Risk-of-bias assessments are summarized in Supplementary File 3. Among the 21 single-arm observational studies assessed using the JBI Critical Appraisal Checklist for Case Series, 8 were judged to be at low risk of bias, 12 had moderate concerns, and 1 conference-abstract-only study had high concerns because of insufficient methodological reporting. Both comparative retrospective cohort studies had moderate concerns, primarily because of baseline differences between treatment groups, non-random treatment allocation, and potential residual confounding. The randomized controlled trial was judged as having some concerns because random-sequence generation and allocation concealment were insufficiently described, blinding was not reported, and some clinical outcomes included subjective components. Detailed item-level assessments for all included studies are provided in Supplementary File 3.

### Study characteristics

The 24 included studies had a combined reported sample size of 1,152 for intravenous polymyxin treatment. Because the original literature was heterogeneous in both study design and reporting format, the unit of analysis varied across studies and included unique patients, treatment episodes, courses, and indications. Most studies were conducted in pediatric intensive care settings; however, some were performed in other high-acuity inpatient settings, including a pediatric burn unit and mixed hospital wards. Most studies evaluated documented resistant Gram-negative infections, whereas some also included empirically treated or highly suspected resistant infections based on local epidemiology and clinical severity. Detailed study characteristics, sample sizes, units of analysis, treatment regimens, adverse effects, and risk-of-bias tools are provided in Supplementary File 4.

Baseline diagnoses and comorbid conditions varied across the included studies. Several cohorts included critically ill children in pediatric intensive care units (PICUs), pediatric burn patients, hematology/oncology patients, and mixed hospitalized pediatric populations. However, detailed comorbidity profiles, including immune status, malignancy, organ dysfunction, and preexisting chronic kidney disease, were not consistently reported across studies. Some studies also involved severe nosocomial or hospital-acquired infections in which concomitant Gram-positive or fungal infections, or the use of additional antimicrobial agents, may have been present, but these data were not reported in a standardized or extractable format. Therefore, subgroup analyses according to underlying diagnosis, comorbidity, co-infection, or chronic renal disease could not be performed.

Some included studies enrolled neonatal patients within broader pediatric cohorts. However, neonatal-specific outcome data were not consistently reported separately in the original publications; therefore, a separate neonatal subgroup meta-analysis was not feasible.

With regard to polymyxin dosing, 18 studies reported doses in milligrams, ranging from 2.5 to 15 mg/kg/day, while 6 studies used international units (IU), with doses ranging from 25,000 to 150,000 IU/kg/day. Most studies reported polymyxins administered in divided doses. One study reported a loading dose of 225,000 IU/kg. Because dose units differed across studies and both colistin/CMS and polymyxin B were represented, dose-related subgroup findings should be interpreted cautiously.

In most studies, polymyxin was used in combination therapy, commonly with carbapenems, glycopeptides, and aminoglycosides. Two studies also reported intravenous polymyxin combined with other administration routes (e.g., nebulized, intraventricular).

### Mortality

A total of 18 studies, with a combined reported sample size of 995, provided data on mortality. The pooled all-cause mortality proportion was 22% (95% CI: 16%–27%; *I*^2^ = 79%; Figure 2). Among the eight studies reporting infection-related mortality, the pooled infection-related mortality proportion was 14% (95% CI: 11%–17%; *I*^2^ = 19%; Figure 3).

**Figure 2:**
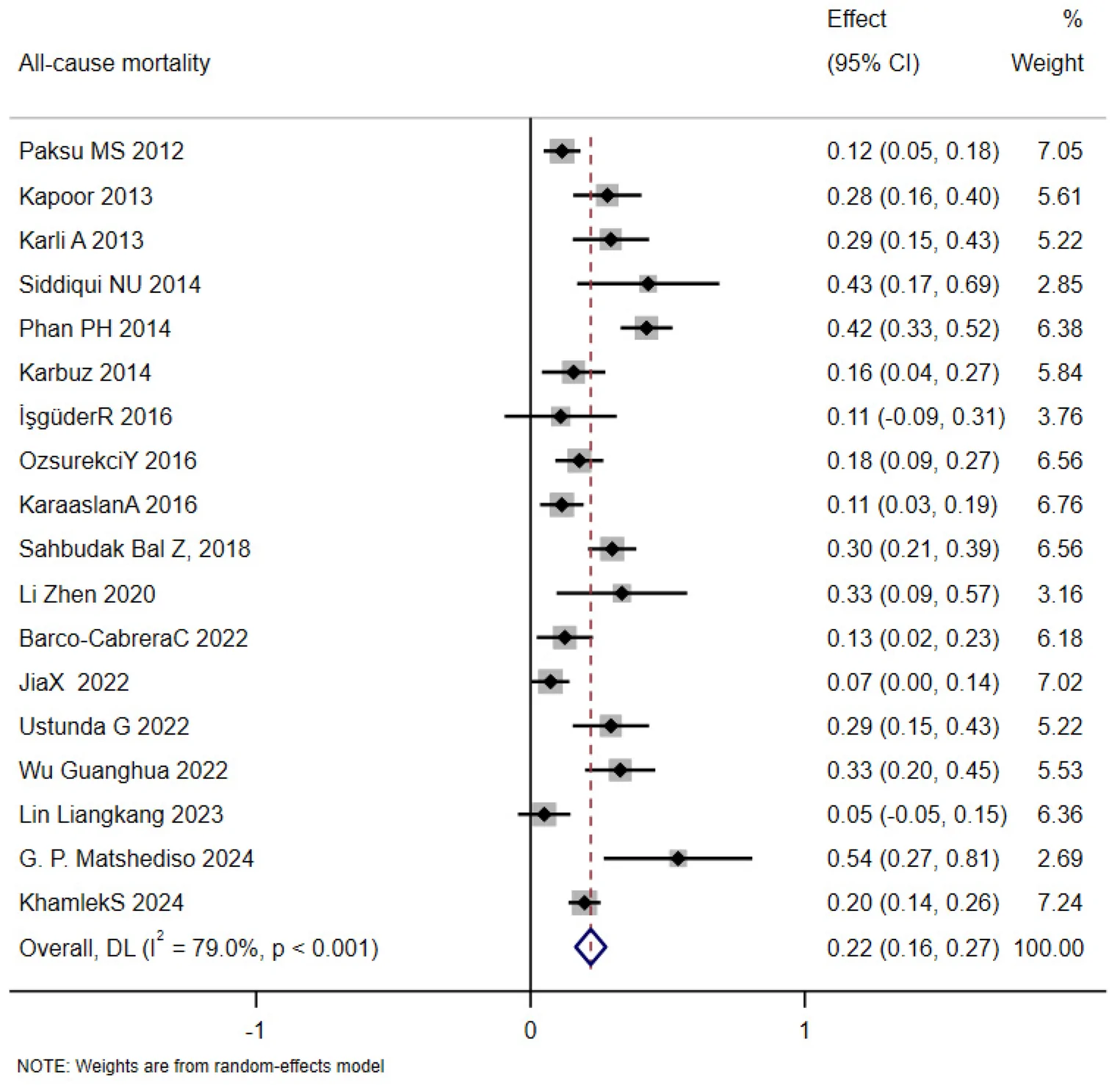
Forest plot of all-cause mortality.

**Figure 3:**
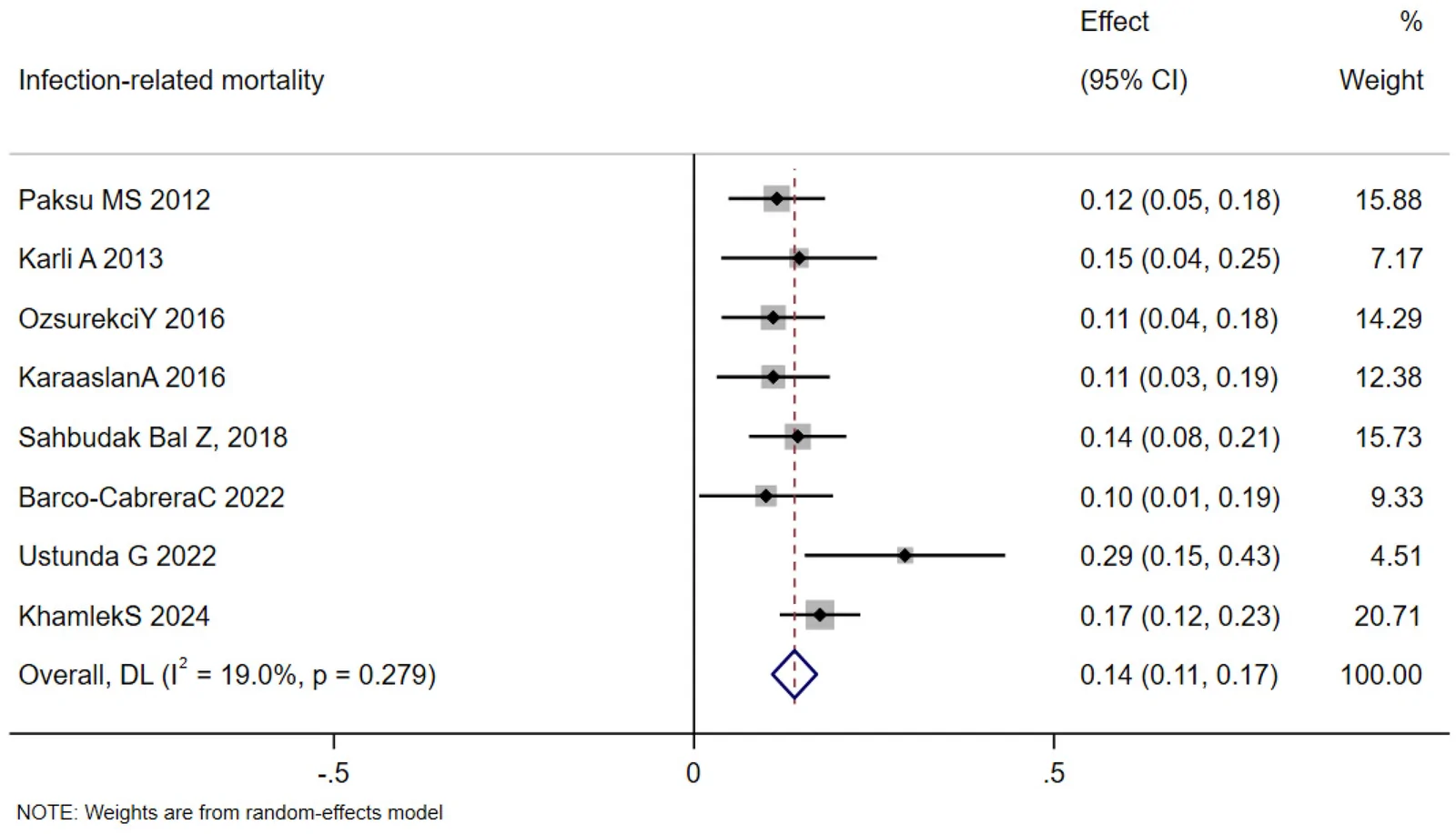
Forest plot of infection-related mortality.

Descriptive subgroup estimates for all-cause mortality were 19% (95% CI: 6%–32%), 21% (95% CI: 14%–29%), and 26% (95% CI: 14%–37%) for reported dose categories <2.5, 2.5–5.0, and >5.0 mg/kg/day, respectively; 24% (95% CI: 17%–30%) for MDR-GNB and 19% (95% CI: 8%–29%) for carbapenem-resistant Gram-negative bacteria (CR-GNB); and 20% (95% CI: 14%–27%) for treatment durations of 7–14 days and 25% (95% CI: 14%–35%) for durations >14 days (Figure 4). Because formal tests for interaction were not reported, these findings were interpreted descriptively rather than as evidence of statistically significant between-subgroup differences.

**Figure 4:**
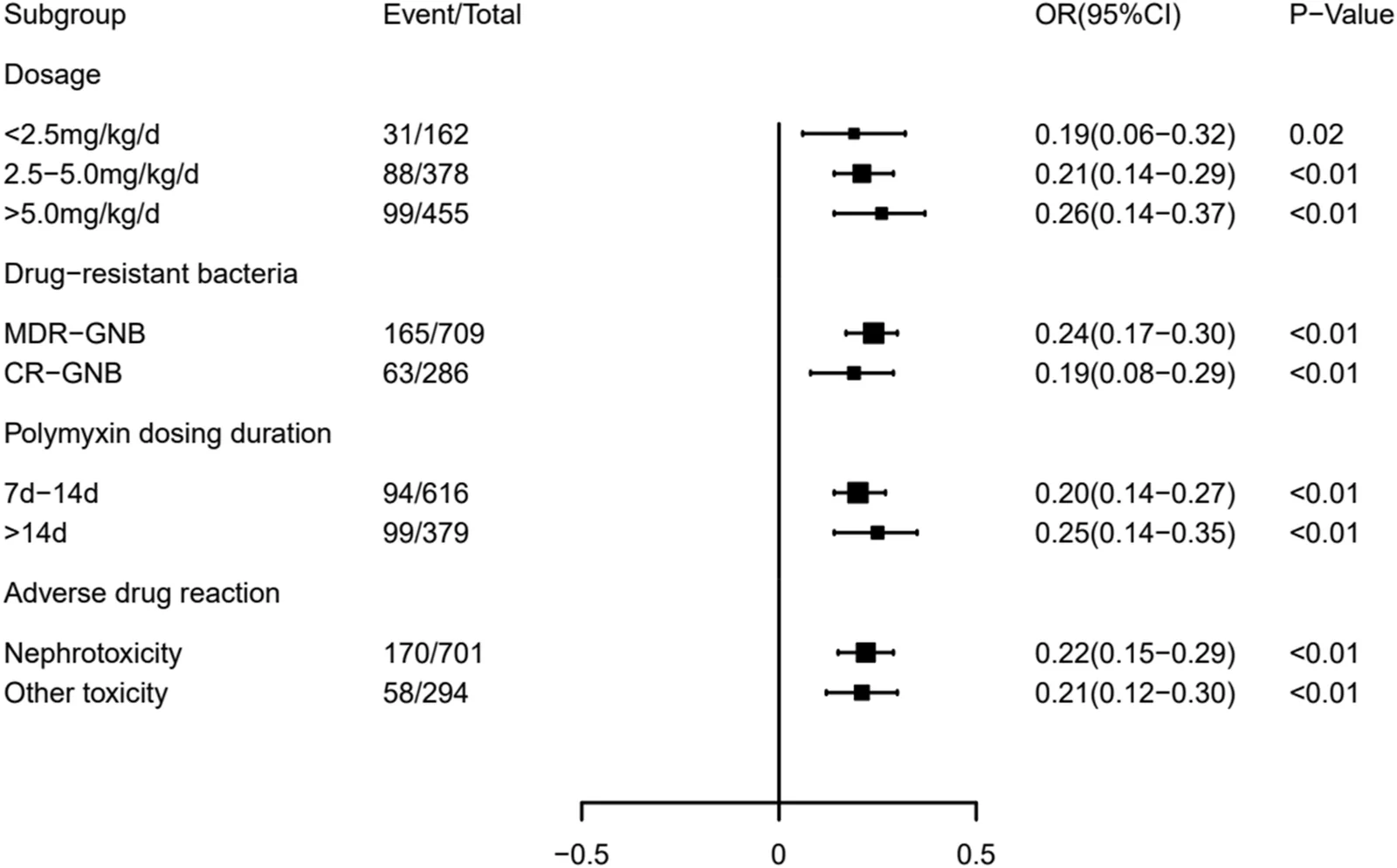
Subgroup analysis of all-cause mortality.

### Clinical improvement

A total of 18 studies, with a combined reported sample size of 995, provided data on clinical improvement outcomes. The pooled clinical improvement proportion was 65% (95% CI: 58%–72%; *I*^2^ = 82.6%; Figure 5).

**Figure 5:**
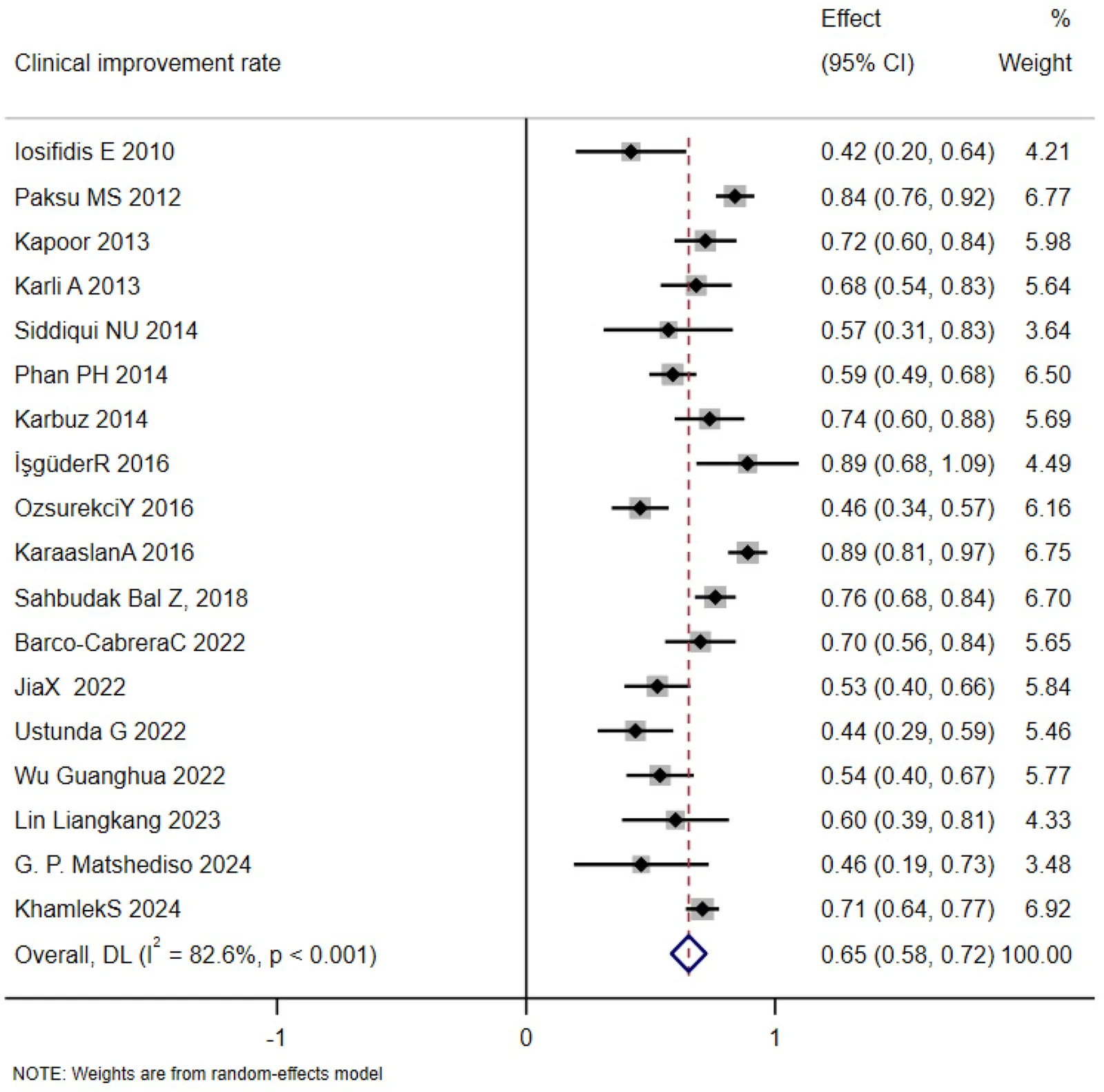
Forest plot of clinical improvement.

### Adverse events

A total of 24 studies comprising 1,152 reported patients or treatment units contributed data on overall adverse events. The pooled overall adverse-event proportion was 13% (95% CI: 9%–17%; *I*^2^ = 84.6%; Figure 6). Nephrotoxicity was the most commonly reported adverse event, with a pooled proportion of 10% (95% CI: 7%–14%; *I*^2^ = 85.1%; Figure 7). Reports of neurotoxicity and hepatotoxicity were uncommon.

**Figure 6:**
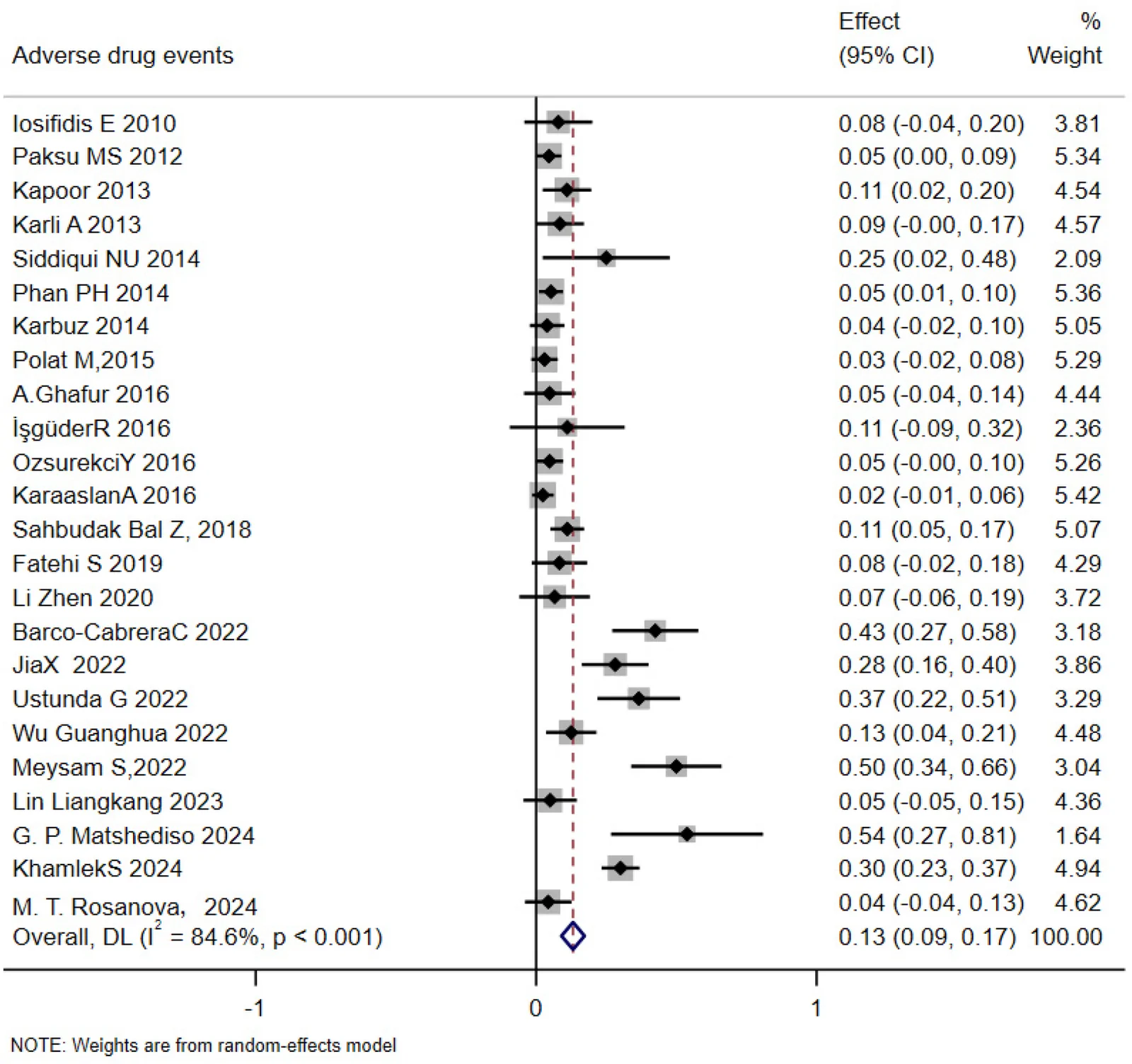
Forest plot of overall adverse events.

**Figure 7:**
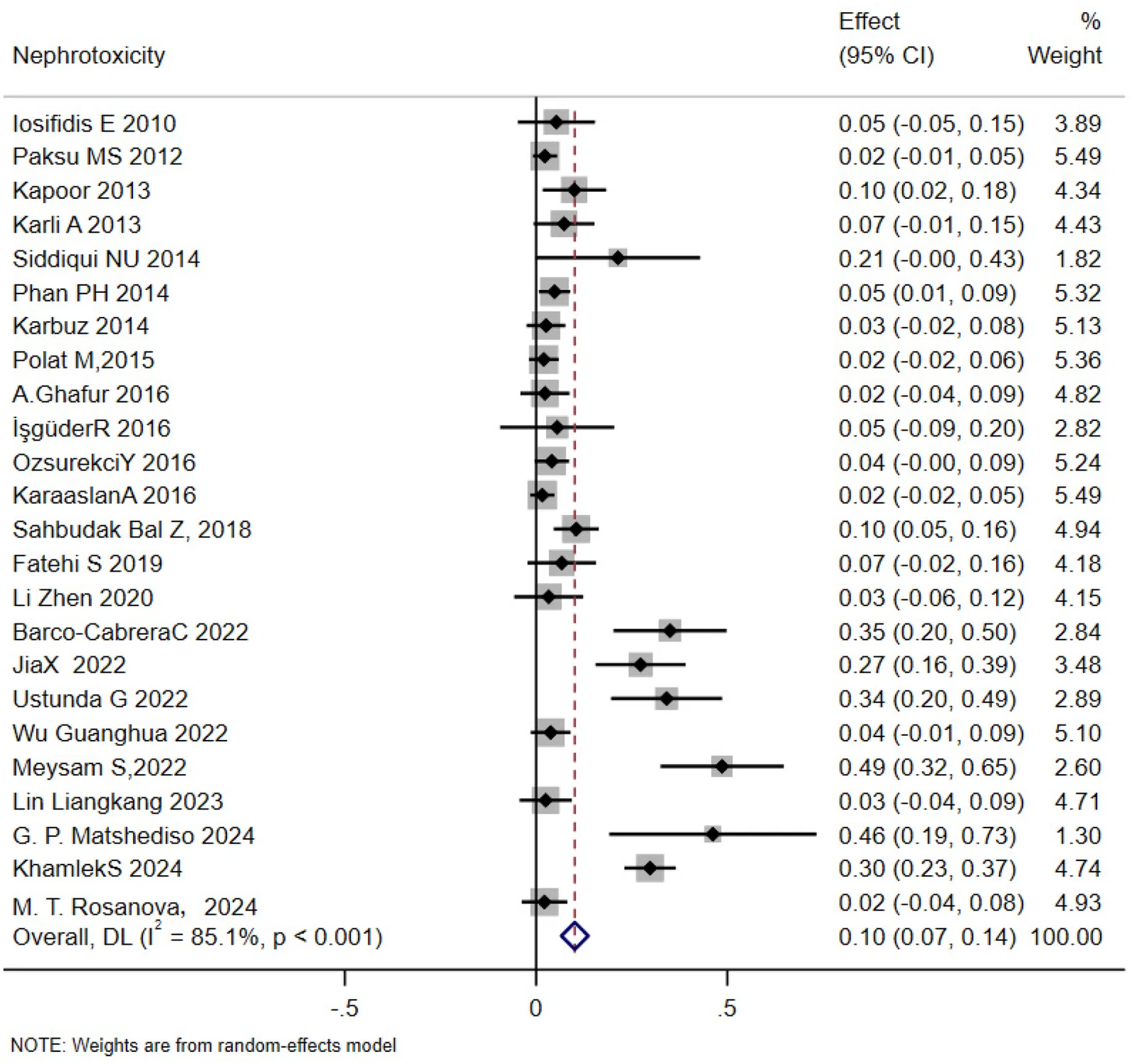
Forest plot of nephrotoxicity.

Nephrotoxicity definitions differed substantially across studies. Some studies used changes in serum creatinine or acute kidney injury criteria, whereas others reported renal adverse events without a detailed operational definition. In addition, baseline renal function, preexisting chronic kidney disease, and exposure to concomitant nephrotoxic agents were inconsistently reported. These factors limited the ability to determine whether nephrotoxicity was directly attributable to polymyxin therapy.

Therefore, the pooled nephrotoxicity estimate should be interpreted as an observed renal adverse-event proportion rather than a causal estimate of polymyxin-induced nephrotoxicity.

### Publication bias

For all-cause mortality, the funnel plot was asymmetric and Egger's test suggested possible small-study effects (*P* = 0.048). The trim-and-fill procedure did not materially change the pooled estimate; however, these analyses should be interpreted cautiously because of the substantial heterogeneity and the limitations of asymmetry tests in meta-analyses of proportions (Figures 8–10).

**Figure 8:**
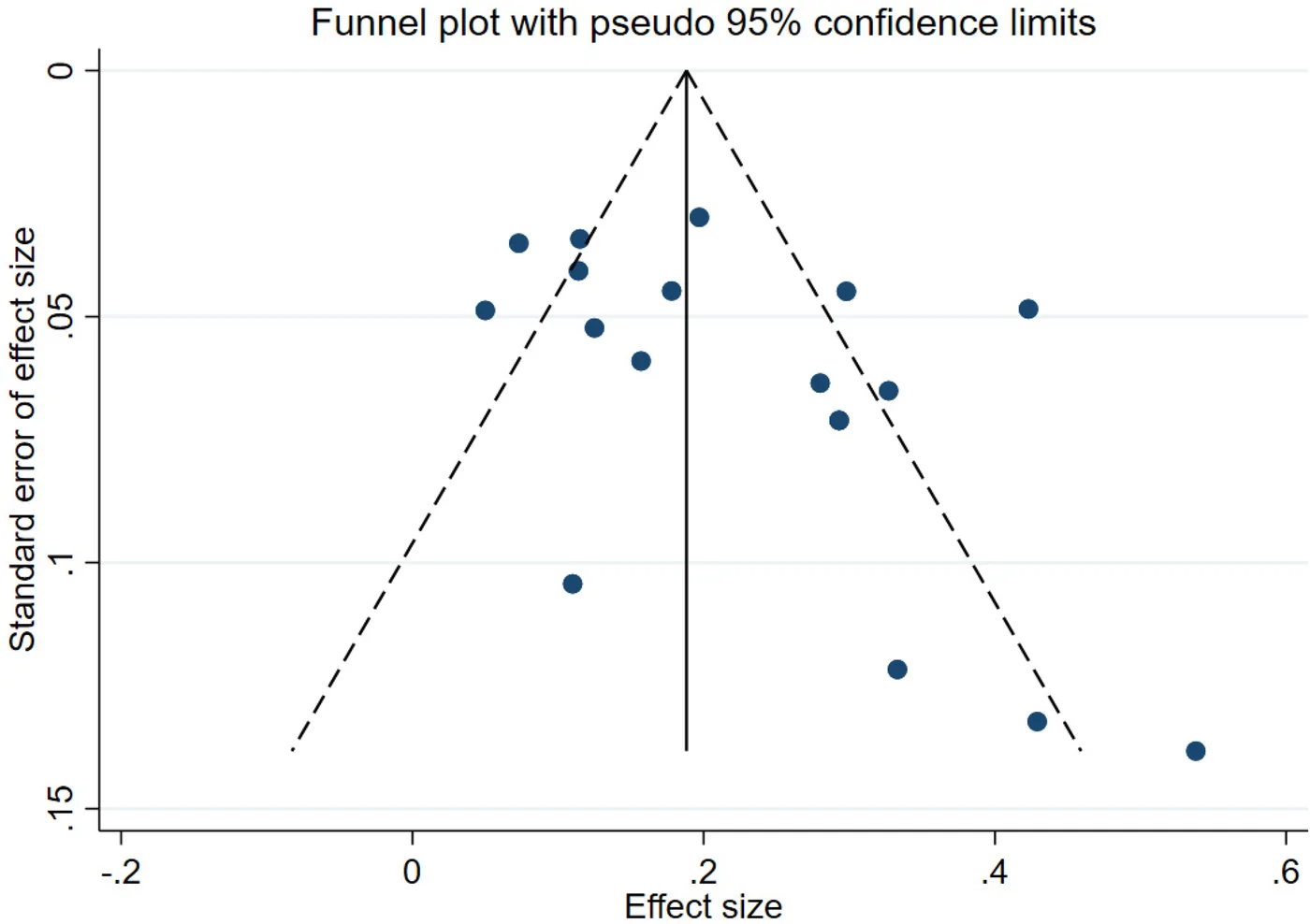
Funnel plot for all-cause mortality.

**Figure 9:**
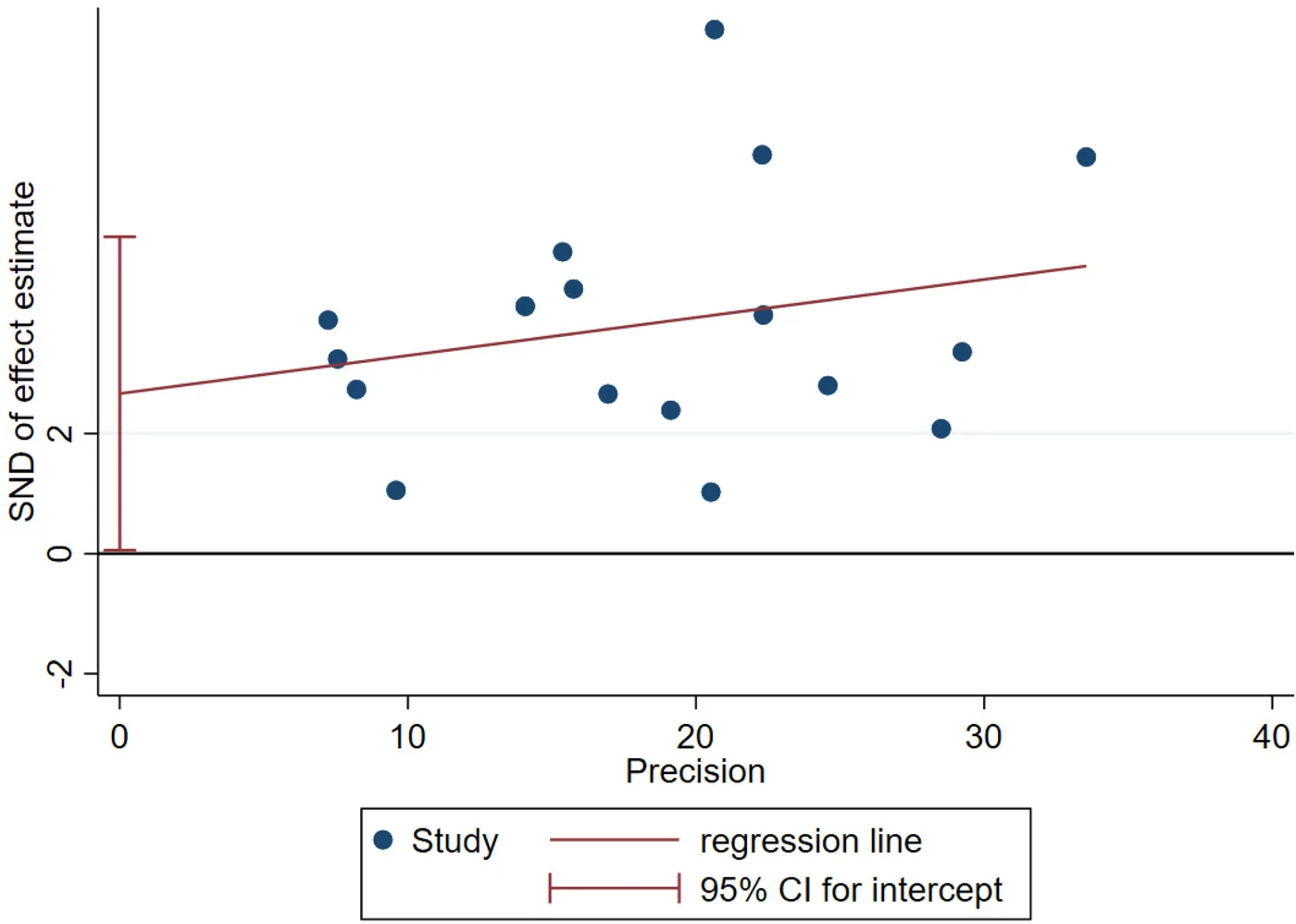
Egger's regression test for small-study effects.

**Figure 10:**
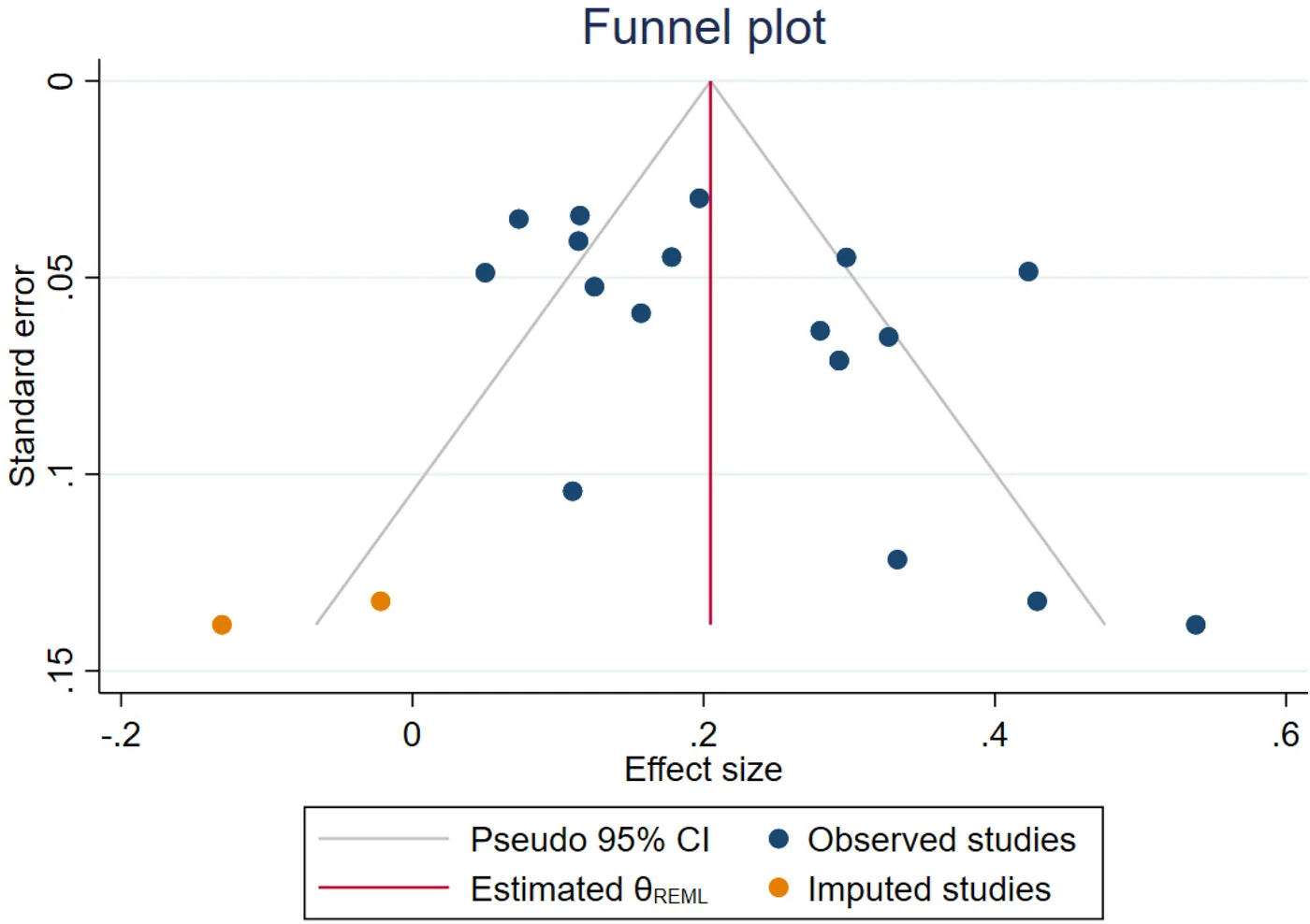
Trim-and-fill analysis for all-cause mortality.

### Sensitivity analysis

Sensitivity analysis based on sequential omission of individual studies showed that the pooled all-cause mortality estimate remained broadly stable (Figure 11).

**Figure 11:**
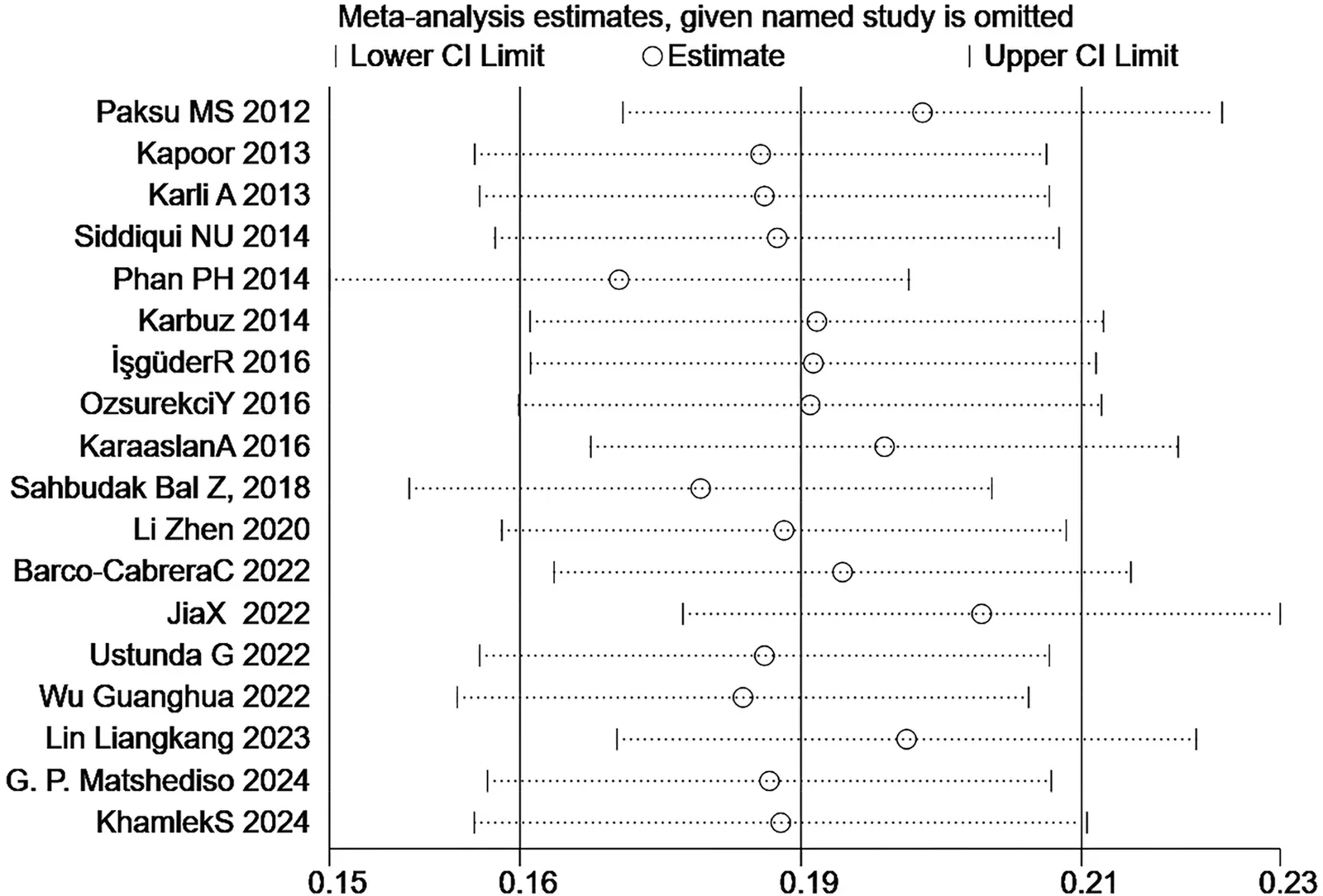
Leave-one-out sensitivity analysis for all-cause mortality.

## Discussion

Resistant Gram-negative bacteria have become an important cause of infection in pediatric settings, leading to increased morbidity and mortality. In this study, 24 studies contributed 1,152 reported patients or treatment units across predominantly high-acuity pediatric inpatient cohorts, including PICU populations, burn unit cohorts, and other hospitalized pediatric settings. Most studies involved MDR-GNB and CR-GNB infections, with *A. baumannii* frequently reported. The pooled all-cause mortality proportion was 22%, the infection-related mortality proportion was 14%, the clinical improvement proportion was 65%, the overall adverse-event proportion was 13%, and the nephrotoxicity proportion was 10%. Neurotoxicity and hepatotoxicity were infrequently reported. Because most studies administered polymyxins concurrently with other medications and lacked an untreated comparator, neither comparative efficacy nor a direct causal link between polymyxin exposure and nephrotoxicity can be established. Descriptive subgroup estimates were numerically higher in studies reporting doses >5.0 mg/kg/day, MDR-GNB infections, and treatment durations >14 days, but formal interaction tests were not reported.

The prognosis of children receiving intravenous polymyxins may also be influenced by baseline diagnosis, comorbidities, immune status, infection severity, and co-infections. For example, children in PICUs, burn units, and hematology/oncology wards may differ substantially in baseline risk of mortality, renal injury, and exposure to additional broad-spectrum antibacterial or antifungal agents. Concomitant Gram-positive or fungal infections, when present, may also affect survival independently of resistant Gram-negative infection. However, these factors were not consistently or sufficiently reported in the included studies. Therefore, the pooled mortality and adverse-event estimates should be interpreted as observed outcomes in heterogeneous hospitalized pediatric populations rather than as outcomes attributable solely to resistant Gram-negative infection or polymyxin exposure.

A previous meta-analysis published in 2019 reported the following outcomes for polymyxin use in critically ill children with multidrug-resistant bacterial infections: all-cause mortality 29.5% (95% CI: 21.7%–38.1%) and infection-related mortality 16.6% (95% CI: 12.2%–21.5%), with nephrotoxicity and neurotoxicity rates of 6.1% and 0.5%, respectively (39). Our study observed somewhat lower pooled mortality estimates and a higher reported nephrotoxicity proportion (10%). These differences may reflect variation in study populations, care settings, pathogen profiles, route and dose reporting, nephrotoxicity definitions, and the inclusion of more recent real-world pediatric cohorts. Therefore, comparisons with previous meta-analyses should be made cautiously.

Compared with the 2019 meta-analysis, the present review updates the literature search to 31 January 2025, includes recently published pediatric cohorts from 2020 to 2024, incorporates both English- and Chinese-language databases, evaluates both colistin/colistimethate sodium and polymyxin B, and includes hospitalized pediatric settings beyond the PICU, such as burn units, hematology/oncology wards, and mixed hospital wards. We also explicitly distinguished patient-level from treatment-level reporting units—including patients, episodes, courses, and indications—to improve transparency in the interpretation of pooled estimates.

Our study found a lower infection-related mortality proportion but a higher nephrotoxicity proportion than the earlier review. This may reflect frequent concomitant use of other nephrotoxic drugs (e.g., aminoglycosides and vancomycin), the high baseline risk of kidney injury in severely ill hospitalized children, and differences in nephrotoxicity definitions. Because most studies did not use multivariable adjustment, the pooled nephrotoxicity estimate should not be interpreted as a causal effect of polymyxin exposure. Standardized definitions, such as kidney disease: improving global outcomes (KDIGO) criteria, should be used in future studies.

Although the clinical improvement rate was relatively high (65% in this study), some studies defined “improvement” based mainly on symptom relief without consistently assessing microbiological clearance, potentially overestimating efficacy. This highlights the importance of consistent outcome definitions in future pediatric studies.

Figure 4 showed numerically higher all-cause mortality proportions in the >5.0 mg/kg/day group (26%) than in the 2.5–5.0 mg/kg/day (21%) and <2.5 mg/kg/day (19%) groups, in MDR-GNB studies (24%) than in CR-GNB studies (19%), and with treatment durations >14 days (25%) than with durations of 7–14 days (20%). These comparisons were descriptive because formal tests for subgroup differences were not reported. The observed patterns may reflect confounding by illness severity, treatment indication, drug formulation, dose unit heterogeneity, and treatment duration rather than direct harmful effects of polymyxins.

Finally, we acknowledge several limitations. First, most included studies were retrospective, and only a small number were prospective or randomized, which limits the certainty of the evidence. Second, the included studies were heterogeneous in clinical setting, encompassing pediatric intensive care units, burn units, and other inpatient wards. Third, although most cohorts involved documented resistant Gram-negative infections, some studies also included empirically treated or highly suspected resistant infections, which may have increased clinical heterogeneity. Fourth, the unit of analysis varied across studies and included unique patients, treatment episodes, courses, and indications; therefore, statistical independence may not have been fully preserved across all pooled estimates. Finally, a small proportion of data was derived from extractable pediatric subgroup analyses within mixed-age cohorts. These factors should be considered when interpreting the pooled results.

Another important limitation is that underlying diagnoses, comorbidities, baseline renal function, chronic kidney disease, concomitant Gram-positive or fungal infections, and concomitant nephrotoxic medications were not consistently reported. These factors may have affected mortality, clinical improvement, and nephrotoxicity, but could not be explored quantitatively. In addition, nephrotoxicity definitions varied across studies, which may have contributed to heterogeneity in the pooled safety estimates. Neonatal patients were included within broader pediatric cohorts in some studies, but neonatal-specific outcomes were not consistently extractable; this limited our ability to compare neonatal and non-neonatal populations separately.

Overall, the current evidence describes outcomes observed during intravenous polymyxin-containing therapy in hospitalized pediatric patients with resistant Gram-negative infections, but it remains insufficient to establish comparative efficacy or causal toxicity.

## Conclusion

Among hospitalized children receiving intravenous polymyxin-containing therapy for resistant Gram-negative infections, the pooled all-cause mortality proportion was 22%, the clinical improvement proportion was 65%, the overall adverse-event proportion was 13%, and the nephrotoxicity proportion was 10%. Because most evidence was uncontrolled and heterogeneous, these estimates describe observed outcomes rather than comparative efficacy or causal toxicity. Prospective multicenter pediatric studies with standardized dosing, outcome definitions, and safety assessment are needed.

## Data Availability

The original contributions presented in the study are included in the article/Supplementary Material; further inquiries can be directed to the corresponding author.
