# Supplementary material for: Clinical outcomes and adverse events associated with intravenous polymyxin-containing therapy in hospitalized children with resistant Gram-negative infections: a systematic review and meta-analysis
Source: Front Pediatr. 2026 Sep 21;14:1928217. doi: 10.3389/fped.2026.1928217 (PMC13635143; doi:10.3389/fped.2026.1928217)
Supplement: Supplementary file 1 [file Table1.docx]

Supplementary Material 1. PRISMA 2020 Checklist

| **Section and topic** | **Item #** | **Checklist item** | Location where item is reported |
| --- | --- | --- | --- |
| TITLE | 1 | Identify the report as a systematic review. | Title |
| ABSTRACT | 2 | See the PRISMA 2020 for Abstracts checklist. | Abstract |
| INTRODUCTION | 3 | Describe the rationale for the review in the context of existing knowledge. | Introduction, paragraphs 1-4 |
| INTRODUCTION | 4 | Provide an explicit statement of the objective(s) or question(s) the review addresses. | Introduction, final paragraph |
| METHODS | 5 | Specify the inclusion and exclusion criteria for the review and how studies were grouped for the syntheses. | Study selection |
| METHODS | 6 | Specify all databases, registers, websites, organisations, reference lists and other sources searched or consulted to identify studies, and the date when each source was last searched or consulted. | Search strategy |
| METHODS | 7 | Present the full search strategies for all databases, registers and websites, including any filters and limits used. | Supplementary Material 2 |
| METHODS | 8 | Specify the methods used to decide whether a study met the inclusion criteria of the review, including how many reviewers screened each record and each report retrieved and whether they worked independently. | Study selection; Literature search; Figure 1 (partially reported) |
| METHODS | 9 | Specify the methods used to collect data from reports, including how many reviewers collected data from each report, whether they worked independently and processes for obtaining or confirming data from study investigators. | Data extraction |
| METHODS | 10a | List and define all outcomes for which data were sought. Specify whether all results that were compatible with each outcome domain were sought and, if not, the methods used to decide which results to collect. | Outcomes and definitions |
| METHODS | 10b | List and define all other variables for which data were sought and describe any assumptions made about any missing or unclear information. | Data extraction |
| METHODS | 11 | Specify the methods used to assess risk of bias in the included studies. | Risk-of-bias assessment; Supplementary Material 3 |
| METHODS | 12 | Specify for each outcome the effect measure(s) used in the synthesis or presentation of results. | Statistical analysis |
| METHODS | 13a | Describe the processes used to decide which studies were eligible for each synthesis. | Study selection; Mortality; Clinical improvement rate; Adverse events (partially reported) |
| METHODS | 13b | Describe any methods required to prepare the data for presentation or synthesis, such as handling of missing summary statistics or data conversions. | Statistical analysis; Study characteristics |
| METHODS | 13c | Describe any methods used to tabulate or visually display results of individual studies and syntheses. | Figures 1-11; Supplementary Materials 3–4 |
| METHODS | 13d | Describe any methods used to synthesize results and provide a rationale for the choice(s). If meta-analysis was performed, describe the model(s), method(s) to identify statistical heterogeneity, and software package(s) used. | Statistical analysis |
| METHODS | 13e | Describe any methods used to explore possible causes of heterogeneity among study results. | Statistical analysis |
| METHODS | 13f | Describe any sensitivity analyses conducted to assess robustness of the synthesized results. | Not included in the submitted version |
| METHODS | 14 | Describe any methods used to assess risk of bias due to missing results in a synthesis (arising from reporting biases). | Statistical analysis; Publication bias |
| METHODS | 15 | Describe any methods used to assess certainty (or confidence) in the body of evidence for an outcome. | Not assessed |
| RESULTS | 16a | Describe the results of the search and selection process, from the number of records identified to the number of studies included in the review, ideally using a flow diagram. | Literature search; Figure 1 |
| RESULTS | 16b | Cite studies that might appear to meet the inclusion criteria, but which were excluded, and explain why they were excluded. | Not individually listed |
| RESULTS | 17 | Cite each included study and present its characteristics. | Study characteristics; Supplementary Material 4 |
| RESULTS | 18 | Present assessments of risk of bias for each included study. | Risk-of-bias assessment; Supplementary Material 3 |
| RESULTS | 19 | For all outcomes, present, for each study: summary statistics for each group (where appropriate) and an effect estimate with its precision. | Supplementary Material 4; Figures 2-7 (partially reported) |
| RESULTS | 20a | For each synthesis, briefly summarise the characteristics and risk of bias among contributing studies. | Study characteristics; Risk-of-bias assessment; Supplementary Materials 3–4 |
| RESULTS | 20b | Present results of all statistical syntheses conducted. If meta-analysis was done, present for each the summary estimate and its precision and measures of statistical heterogeneity. | Mortality; Clinical improvement rate; Adverse events; Figures 2-7 |
| RESULTS | 20c | Present results of all investigations of possible causes of heterogeneity among study results. | Subgroup analysis; Figure 4 |
| RESULTS | 20d | Present results of all sensitivity analyses conducted to assess the robustness of the synthesized results. | Not included in the submitted version |
| RESULTS | 21 | Present assessments of risk of bias due to missing results (arising from reporting biases) for each synthesis assessed. | Publication bias; Figures 8-10 |
| RESULTS | 22 | Present assessments of certainty (or confidence) in the body of evidence for each outcome assessed. | Not assessed |
| DISCUSSION | 23a | Provide a general interpretation of the results in the context of other evidence. | Discussion, paragraphs 1-5 |
| DISCUSSION | 23b | Discuss any limitations of the evidence included in the review. | Discussion, limitations paragraph |
| DISCUSSION | 23c | Discuss any limitations of the review processes used. | Discussion, limitations paragraph (partially reported) |
| DISCUSSION | 23d | Discuss implications of the results for practice, policy, and future research. | Discussion, final paragraphs; Conclusion |
| OTHER | 24a | Provide registration information for the review, including register name and registration number, or state that the review was not registered. | Materials and Methods, first paragraph |
| OTHER | 24b | Indicate where the review protocol can be accessed, or state that a protocol was not prepared. | PROSPERO registration reported in Materials and Methods; protocol access not separately reported |
| OTHER | 24c | Describe and explain any amendments to information provided at registration or in the protocol. | Not reported |
| OTHER | 25 | Describe sources of financial or non-financial support for the review, and the role of the funders or sponsors. | Funding |
| OTHER | 26 | Declare any competing interests of review authors. | Competing interests |
| OTHER | 27 | Report which of the following are publicly available and where they can be found: template data collection forms, data extracted from included studies, data used for analyses, analytic code, and any other materials used in the review. | Availability of data and materials; Additional files |
