# Supplementary material for: Clinical outcomes and adverse events associated with intravenous polymyxin-containing therapy in hospitalized children with resistant Gram-negative infections: a systematic review and meta-analysis
Source: Front Pediatr. 2026 Sep 21;14:1928217. doi: 10.3389/fped.2026.1928217 (PMC13635143; doi:10.3389/fped.2026.1928217)
Supplement: Supplementary file 2 [file Table2.docx]

Supplementary Material 2. Complete Search Strategies

Clinical Outcomes and Adverse Events Associated with Intravenous Polymyxin-Containing Therapy in Hospitalized Children with Resistant Gram-Negative Infections: A Systematic Review and Meta-Analysis

Databases searched: PubMed, EMBASE, Web of Science Core Collection, Cochrane Library, CNKI, Wanfang Data, SinoMed, and VIP.

Coverage period: database inception to 31 January 2025.

The search strategy combined terms for polymyxins/colistin and pediatric populations. Database-specific syntax was adapted as appropriate. Reference lists of relevant studies and reviews were also screened manually.

# PubMed

(("Polymyxins"[Mesh] OR polymyxin*[Title/Abstract] OR "polymyxin B"[Title/Abstract]

OR colistin[Title/Abstract] OR colistimethate[Title/Abstract]

OR "colistimethate sodium"[Title/Abstract]

OR "colistin methanesulfonate"[Title/Abstract])

AND

("Infant"[Mesh] OR "Child"[Mesh] OR "Adolescent"[Mesh]

OR pediatric*[Title/Abstract] OR paediatric*[Title/Abstract]

OR child*[Title/Abstract] OR infant*[Title/Abstract]

OR neonat*[Title/Abstract] OR newborn*[Title/Abstract]

OR adolescen*[Title/Abstract] OR teen*[Title/Abstract]))

# EMBASE

('polymyxin'/exp OR polymyxin*:ti,ab,kw OR 'polymyxin b':ti,ab,kw

OR colistin:ti,ab,kw OR colistimethate:ti,ab,kw

OR 'colistimethate sodium':ti,ab,kw

OR 'colistin methanesulfonate':ti,ab,kw)

AND

('infant'/exp OR 'child'/exp OR 'adolescent'/exp

OR pediatric*:ti,ab,kw OR paediatric*:ti,ab,kw

OR child*:ti,ab,kw OR infant*:ti,ab,kw

OR neonat*:ti,ab,kw OR newborn*:ti,ab,kw

OR adolescen*:ti,ab,kw OR teen*:ti,ab,kw)

# Web of Science

TS=((polymyxin* OR "polymyxin B" OR colistin OR colistimethate

OR "colistimethate sodium" OR "colistin methanesulfonate")

AND

(pediatric* OR paediatric* OR child* OR infant*

OR neonat* OR newborn* OR adolescen* OR teen*))

# Cochrane Library

((polymyxin* OR "polymyxin B" OR colistin OR colistimethate

OR "colistimethate sodium" OR "colistin methanesulfonate")

AND

(pediatric* OR paediatric* OR child* OR infant*

OR neonat* OR newborn* OR adolescen* OR teen*)):ti,ab,kw

# CNKI

TS=((polymyxin* OR "polymyxin B" OR colistin

OR colistimethate OR "colistimethate sodium")

AND

(pediatric* OR paediatric* OR child* OR infant*

OR neonat* OR newborn* OR adolescen*))

# Wanfang Data

Topic: ((polymyxin* OR "polymyxin B" OR colistin

OR colistimethate OR "colistimethate sodium")

AND

(pediatric* OR paediatric* OR child* OR infant*

OR neonat* OR newborn* OR adolescen*))

# SinoMed

((polymyxin* OR "polymyxin B" OR colistin

OR colistimethate OR "colistimethate sodium")

AND

(pediatric* OR paediatric* OR child* OR infant*

OR neonat* OR newborn* OR adolescen*))

# VIP

Title/Keyword:

((polymyxin* OR "polymyxin B" OR colistin

OR colistimethate OR "colistimethate sodium")

AND

(pediatric* OR paediatric* OR child* OR infant*

OR neonat* OR newborn* OR adolescen*))
