# Supplementary material for: Clinical outcomes and adverse events associated with intravenous polymyxin-containing therapy in hospitalized children with resistant Gram-negative infections: a systematic review and meta-analysis
Source: Front Pediatr. 2026 Sep 21;14:1928217. doi: 10.3389/fped.2026.1928217 (PMC13635143; doi:10.3389/fped.2026.1928217)
Supplement: Supplementary file 3 [file Table3.docx]

Supplementary Material 3. Risk-of-Bias Assessment of the Included Studies

Risk of bias was assessed using Cochrane RoB 2 for the randomized trial [10], the JBI Critical Appraisal Checklist for Cohort Studies for comparative cohort studies [11], and the JBI Critical Appraisal Checklist for Case Series for single-arm observational studies [12]. Judgments were made conservatively from the information reported in the published articles. Y = Yes; N = No; U = Unclear because reporting was insufficient; NA = Not applicable. The overall categories are review-specific summary judgments and are not official numerical JBI scores.

**Table S1A. JBI Critical Appraisal Checklist for Case Series [12]**

| **Study** | **Q1** | **Q2** | **Q3** | **Q4** | **Q5** | **Q6** | **Q7** | **Q8** | **Q9** | **Q10** | **Overall** |
| --- | --- | --- | --- | --- | --- | --- | --- | --- | --- | --- | --- |
| Iosifidis et al. (2010) | Y | Y | Y | U | U | Y | Y | Y | Y | Y | Moderate |
| Paksu et al. (2012) | Y | Y | Y | U | U | Y | Y | Y | Y | Y | Moderate |
| Kapoor et al. (2013) | Y | Y | Y | U | U | Y | Y | Y | Y | Y | Moderate |
| Karli et al. (2013) | Y | Y | Y | U | U | Y | Y | Y | Y | Y | Moderate |
| Siddiqui et al. (2014) | Y | Y | Y | Y | Y | Y | Y | Y | Y | Y | Low |
| Phan and Nguyen (2014) | U | U | U | U | U | Y | U | Y | Y | U | High* |
| Karbuz et al. (2014) | Y | Y | Y | U | U | Y | Y | Y | Y | Y | Moderate |
| Ghafur et al. (2016) | Y | Y | Y | U | U | Y | Y | Y | Y | Y | Moderate |
| İşgüder et al. (2016) | Y | Y | Y | U | U | Y | Y | Y | Y | U | Moderate |
| Karaaslan et al. (2016) | Y | Y | Y | Y | Y | Y | Y | Y | Y | Y | Low |
| Sahbudak Bal et al. (2018) | Y | Y | Y | U | Y | Y | Y | Y | Y | Y | Moderate |
| Li et al. (2020) | Y | Y | Y | U | U | Y | Y | Y | Y | U | Moderate |
| Barco-Cabrera et al. (2022) | Y | Y | Y | Y | Y | Y | Y | Y | Y | Y | Low |
| Jia et al. (2022) | Y | Y | Y | Y | Y | Y | Y | Y | Y | Y | Low |
| Ustundag et al. (2022) | Y | Y | Y | Y | Y | Y | Y | Y | Y | Y | Low |
| Wu et al. (2022) | Y | Y | Y | U | U | Y | Y | Y | Y | Y | Moderate |
| Meysam et al. (2022) | Y | Y | Y | Y | Y | Y | Y | Y | Y | Y | Low |
| Lin et al. (2023) | Y | Y | Y | U | U | Y | Y | Y | Y | U | Moderate |
| Matshediso et al. (2024) | Y | Y | Y | Y | Y | U | U | Y | Y | U | Moderate |
| Khamlek et al. (2024) | Y | Y | Y | Y | Y | Y | Y | Y | Y | Y | Low |
| Rosanova et al. (2024) | Y | Y | Y | Y | Y | Y | Y | Y | Y | Y | Low |

Case-series domains: Q1, clear inclusion criteria; Q2, condition measured in a standard and reliable way; Q3, valid methods used to identify the condition; Q4, consecutive inclusion; Q5, complete inclusion; Q6, clear demographic reporting; Q7, clear clinical information; Q8, outcomes/follow-up clearly reported; Q9, site/clinic demographics clearly reported; Q10, appropriate statistical analysis.

*Phan and Nguyen (2014) was available only as a conference abstract/brief report; therefore, several domains could not be assessed and the study was judged at high risk because of insufficient reporting.

**Table S1B. JBI Critical Appraisal Checklist for Cohort Studies [11]**

| **Study** | **Q1** | **Q2** | **Q3** | **Q4** | **Q5** | **Q6** | **Q7** | **Q8** | **Q9** | **Q10** | **Q11** | **Q12** | **Overall** |
| --- | --- | --- | --- | --- | --- | --- | --- | --- | --- | --- | --- | --- | --- |
| Polat et al. (2015) | Y | Y | Y | Y | Y | N | Y | Y | Y | Y | NA | Y | Moderate |
| Ozsurekci et al. (2016) | N | Y | Y | Y | Y | N | Y | Y | Y | Y | NA | Y | Moderate |

Cohort domains: Q1, groups similar and recruited from the same population; Q2, exposure assignment measured similarly; Q3, exposure measured validly and reliably; Q4, confounders identified; Q5, strategies to address confounding stated; Q6, participants free of the outcome at baseline; Q7, outcomes measured validly and reliably; Q8, follow-up time sufficient; Q9, follow-up complete; Q10, reasons for incomplete follow-up described; Q11, strategies to address incomplete follow-up; Q12, appropriate statistical analysis. For both comparative cohorts, residual confounding related to treatment selection remained a major concern.

**Table S1C. Cochrane RoB 2 Assessment for the Randomized Trial [10]**

| **Study** | **D1** | **D2** | **D3** | **D4** | **D5** | **Overall judgment** |
| --- | --- | --- | --- | --- | --- | --- |
| Fatehi et al. (2019) | Some concerns | Some concerns | Low risk | Some concerns | Some concerns | Some concerns |

RoB 2 domains: D1, bias arising from the randomization process; D2, bias due to deviations from intended interventions; D3, bias due to missing outcome data; D4, bias in measurement of the outcome; D5, bias in selection of the reported result. Random-sequence generation and allocation concealment were insufficiently described, blinding was not reported, and the clinical outcomes included subjective components; therefore, the overall judgment was “Some concerns.”

**Summary**

Among the 24 included studies, eight single-arm studies were judged at low risk of bias, twelve single-arm studies and both comparative cohort studies had moderate concerns, one abstract-only study was judged at high risk because of insufficient reporting, and the randomized trial was judged as having some concerns. The most frequent limitations were unclear consecutive or complete inclusion, retrospective data collection, limited control of confounding, and incomplete reporting of selection procedures.

Overall-category rule used in this review: for JBI case series, Low = 8–10 “Yes” responses without a “No” in Q1–Q3 or Q8; Moderate = 5–7 “Yes” responses or unresolved selection/reporting concerns; High = ≤4 “Yes” responses or reporting too limited for reliable appraisal. For cohort studies, the overall judgment additionally emphasized baseline comparability and control of confounding. These thresholds were prespecified by the review team for summary presentation and are not official JBI scoring cutoffs.

**References**

[10] Sterne, J.A.C.; Savović, J.; Page, M.J.; Elbers, R.G.; Blencowe, N.S.; Boutron, I.; Cates, C.J.; Cheng, H.Y.; Corbett, M.S.; Eldridge, S.M.; et al. RoB 2: A revised tool for assessing risk of bias in randomised trials. BMJ 2019, 366, l4898.

[11] Barker, T.H.; Stone, J.C.; Sears, K.; Klugar, M.; Tufanaru, C.; Leonardi-Bee, J.; Aromataris, E.; Munn, Z. The revised JBI critical appraisal tool for the assessment of risk of bias for cohort studies. JBI Evid. Synth. 2025, 23, 441–453.

[12] Munn, Z.; Barker, T.H.; Moola, S.; Tufanaru, C.; Stern, C.; McArthur, A.; Stephenson, M.; Aromataris, E. Methodological quality of case series studies: An introduction to the JBI critical appraisal tool. JBI Evid. Synth. 2020, 18, 2127–2133.
