# Supplementary material for: Clinical outcomes and adverse events associated with intravenous polymyxin-containing therapy in hospitalized children with resistant Gram-negative infections: a systematic review and meta-analysis
Source: Front Pediatr. 2026 Sep 21;14:1928217. doi: 10.3389/fped.2026.1928217 (PMC13635143; doi:10.3389/fped.2026.1928217)
Supplement: Supplementary file 4 [file Table4.docx]

Supplementary Material 4. Characteristics of the Included Studies (Part 1 of 2)

| **First author (Year)** | **Mean age/Median age** | **Unit of analysis** | **Sample size** | **Country** | **Study design** | **Pathogens** | **Route of administration** | **Drug and dosage** | **Treatment duration / assessment timing** | **Adverse effects** | **Risk-of-bias tool** |
| --- | --- | --- | --- | --- | --- | --- | --- | --- | --- | --- | --- |
| Iosifidis, E. 2010 | 5 years | patient/course | 13 | Greece | Retrospective study | MDR-GNB | Intravenous, intraventricular, inhalation | Colistin: 40,000–225,000 IU/kg/day | 25 d | Nephrotoxicity | JBI case series |
| Paksu MS 2012 | 2.5 years | patient/episode | 79 | Turkey | Retrospective study | MDR-GNB | Intravenous | Colistin: 5.4 ± 0.6 mg/kg/day | 17.2 d | Nephrotoxicity, neurotoxicity | JBI case series |
| Kapoor 2013 | 3 years | patient | 50 | India | Retrospective study | MDR-GNB | Intravenous | Colistin: 50,000–75,000 IU/kg/day | 14.3 d | Nephrotoxicity | JBI case series |
| Karli A 2013 | 3 years | patient/episode | 31 | Turkey | Retrospective study | MDR-GNB | Intravenous | Colistin: 4.9 ± 0.5 mg/kg/day | 19.8 d | Nephrotoxicity | JBI case series |
| Siddiqui NU 2014 | 3.4 years | patient | 14 | Pakistan | Retrospective study | MDR-GNB | Intravenous | Polymyxin B: 40,000 IU/kg/day (≈4 mg/kg/day) | 8.5 d | Nephrotoxicity | JBI case series |
| Phan PH 2014 | 4 months | patient/episode | 104 | Vietnam | Retrospective study | MDR-GNB | Intravenous | Colistimethate sodium: 6.2 ± 1.2 mg/kg/day | 17 d | Nephrotoxicity | Abstract-only report |
| Karbuz 2014 | 17 months | patient/course | 29 | Turkey | Prospective study | MDR-GNB | Intravenous | Colimycin: 5.0 mg/kg/day | 12 d | Nephrotoxicity | JBI case series |
| Polat M 2015 | 1 month–18 years | patient | 32 | Turkey | Retrospective study | GNB | Intravenous | Colistin: 2.5–5 mg/kg/day | 16 d | Nephrotoxicity | JBI cohort |
| A. Ghafur 2016 | 9 years | patient | 21 | India | Retrospective study | CR-GNB | Intravenous | Colistin: 11.28 mg/kg/day | 7.85 d | Nephrotoxicity | JBI case series |
| Isguder R 2016 | 34 months | patient | 9 | Turkey | Retrospective study | MDR-GNB | Intravenous | Colistin: 4.1 ± 0.7 mg/kg/day | 18.5 d | None | JBI case series |
| Ozsurekci Y 2016 | 29.8 months | patient/course | 73 | Turkey | Retrospective study | MDR-GNB/XDR-GNB | Intravenous | Colistin: 5–10 mg/kg/day | 17 d | Nephrotoxicity | JBI cohort |
| Karaaslan A 2016 | 12 months | patient | 61 | Turkey | Retrospective study | MDR-GNB | Intravenous | Colistin: 5 mg/kg/day | 12 d | Nephrotoxicity, hepatotoxicity, diarrhea | JBI case series |

Supplementary Material 4. Characteristics of the Included Studies (Part 2 of 2)

| **First author (Year)** | **Mean age/Median age** | **Unit of analysis** | **Sample size** | **Country** | **Study design** | **Pathogens** | **Route of administration** | **Drug and dosage** | **Treatment duration / assessment timing** | **Adverse effects** | **Risk-of-bias tool** |
| --- | --- | --- | --- | --- | --- | --- | --- | --- | --- | --- | --- |
| Sahbudak Bal Z 2018 | 55.9 months | patient | 104 | Turkey | Retrospective study | CR-GNB | Intravenous | Colistin: 5 mg/kg/day | 12.5 d | Nephrotoxicity | JBI case series |
| Fatehi S 2019 | 42 months | patient | 30 | Iran | Randomized controlled trial | POS | Intravenous | Loading-dose versus conventional-dose colistin regimen | 72 h efficacy assessment | Nephrotoxicity | RoB 2 |
| Li Zhen 2020 | 6.5 months | patient | 17 | China | Retrospective study | CR-GNB | Intravenous | Colistin: 1.5–3.0 mg/kg/day | 15.6 d | None | JBI case series |
| Barco-Cabrera C 2022 | 10 years | patient/episode | 40 | Colombia | Retrospective study | CR-GNB | Intravenous | Polymyxin B: 25,000 IU/kg/day; colistin: 4 mg CBA/kg/day | 8.5 d | Nephrotoxicity, neurotoxicity | JBI case series |
| Jia X 2022 | 10 years | patient | 55 | China | Retrospective study | CR-GNB | Intravenous | Polymyxin B: 1.5–4.0 mg/kg/day | 10 d | Nephrotoxicity | JBI case series |
| Ustundag G 2022 | 26 months | patient/case | 37 | Turkey | Retrospective study | MDR-GNB | Intravenous | Colistin: 4.2 mg/kg/day | 14 d | Nephrotoxicity, neurotoxicity | JBI case series |
| Wu Guanghua 2022 | 12 months | patient | 52 | China | Retrospective study | CR-GNB | Intravenous | Polymyxin B: 1.5–4.0 mg/kg/day | 13.9 ± 5.9 d | Nephrotoxicity, cutaneous pigmentation | JBI case series |
| Meysam S 2022 | 4.5 months | patient | 37 | Iran | Prospective study | CR-GNB/XDR | Intravenous | Colistin: 4.4 mg/kg/day | 18.94 ± 14.03 d | Nephrotoxicity | JBI case series |
| Lin Liangkang 2023 | 3.4 years | patient | 20 | China | Retrospective study | CR-GNB | Intravenous / nebulized inhalation | Polymyxin B: 3.2 mg/kg/day | 14.0 d | None | JBI case series |
| G. P. Matshediso 2024 | 31.8 months | pediatric subgroup patient | 13 | South Africa | Retrospective study | MDR-GNB | Intravenous | Colistin: 7.5–15 mg/kg/day | 9.2 d | Nephrotoxicity, neurotoxicity | JBI case series |
| Khamlek S 2024 | 3.4 years | patient | 178 | Thailand | Retrospective study | MDR-GNB | Intravenous | Colistin: 5.1 mg/kg/day | 10 d | Nephrotoxicity | JBI case series |
| M. T. Rosanova 2024 | 6 years | patient/indication | 15 | Argentina | Prospective study | MDR-GNB | Intravenous | Colistin: 4.0–7.5 mg/kg/day | 10 d | None | JBI case series |
